# Development and validation of a scale assessing achievement goals in driving

**DOI:** 10.1371/journal.pone.0230349

**Published:** 2020-03-12

**Authors:** Nicolas Mascret, Martin Nicolleau, Isabelle Ragot-Court

**Affiliations:** 1 Aix Marseille Univ, CNRS, ISM, Marseille, France; 2 TS2-LMA, Univ Gustave Eiffel, IFSTTAR, Salon de Provence, France; Tsinghua University, CHINA

## Abstract

Achievement goals have been a major topic of research for more than 30 years. Achievement goals represent what and why individuals want to achieve. This literature has provided a large body of research in many domains (e.g., education, sports, work), but no study has hitherto been conducted in the driving domain. Moreover, no scale was available to assess achievement goals in driving even though driving is an achievement context. Indeed, drivers’ personal competence is engaged and continuously evaluated both by others and the drivers themselves. The present study seeks to fill these gaps. The aims of the study were to emphasize the interest of investigating achievement goals in car driving, to develop and validate a scale named Achievement Goal Questionnaire in Driving (AGQ-D), to compare this baseline model with five alternative models, to assess the gender invariance of the scale, and to study its concurrent validity using interest and self-efficacy in driving, accidents, at-fault accidents, emergency maneuvers, and fines. The results of the Confirmatory Factor Analysis showed the good psychometric properties of the scale completed by 420 French car drivers, in comparison with five alternative models. The scale was also invariant across gender. Finally, the results of the hierarchical regression analyses showed its concurrent validity. The most significant results highlighted that mastery-avoidance goals (i.e., to avoid being a bad driver and avoiding failing in driving task demands) negatively predicted self-reported accidents and at-fault accidents. Performance-approach goals (i.e., to outperform other drivers) also positively predicted self-reported emergency maneuvers. The AGQ-D is now a tool available to develop research in the driving domain and to extend the numerous advances already found in other domains.

## Introduction

Six million traffic accidents and 37,461 fatalities were recorded in 2016 by the police in the United States [1]. In 2018, 3,503 road fatalities were reported in France, with 1,647 road fatalities concerning car drivers [2]. Many studies have highlighted that the human factor was crucial in most traffic accidents involving all types of vehicles [3, 4]. In line with the driving context, investigating the cognitive and psychological characteristics that govern drivers’ action is necessary to better explain the occurrence of crashes [5]. On a broader level, examining drivers’ psychological characteristics or traits, which are more stable, is also a worthwhile perspective to better understand and to improve road safety. Self-reported driving behaviors were linked to many psychological characteristics or traits such as trait anxiety, Big Five personality factors, anger, or decision-making style [6]. Surprisingly, achievement goal theory has not been hitherto used in the driving domain, although this context may be considered an achievement context and although this theoretical framework has, over the last 30 years, produced a very significant volume of research leading to a better understanding of achievement motivation and human behavior [7]. The aim of the present study was to fill this gap.

Achievement motivation leads to the triggering and direction of behaviors oriented toward demonstrating competence or avoiding demonstrating incompetence. Achievement-oriented behaviors are produced when an individual considers his or her performance to be evaluated. In line with these initial considerations, achievement goals are defined as “cognitive representation(s) of a competence-based possibility that an individual seeks to attain” (p. 628) [8]. The first works on achievement goals led to the development of the dichotomous model of achievement goals [9–11]. An individual adopting mastery goals wants to demonstrate competence through task mastery and personal improvement (e.g., in the driving domain, an individual who seeks to master the driving task and to improve his/her driving skills), while an individual adopting performance goals wants to demonstrate competence compared with others (e.g., in the driving domain, an individual who seeks to outperform other drivers). Measures of these two achievement goals were first developed in the education domain [12] and then in the sports domain [13]. A decade later, the trichotomous model of achievement goals was proposed following the works of Elliot and Harackiewicz [14], which included in the dichotomous model the distinction between approach motivation (i.e., the aim of the individual is to approach success) and avoidance motivation (i.e., the aim of the individual is to avoid failure). Performance goals were consequently divided into performance-approach goals (doing well compared to others) and performance-avoidance goals (not doing poorly compared to others). Mastery goals remained the same. In the driving domain, performance-approach goals may be pursued by individuals who want to prove that they are better drivers than others, while performance-avoidance goals may be pursued by individuals who do not want to be identified as worse drivers than others. Measures were developed in the education [15], work [16], and sports [17] domains. Then, a fourth goal was added to the trichotomous model and led to the 2 x 2 achievement goal model [8], crossing two definitions (mastery versus performance) and two valences (approach versus avoidance) of competence. Mastery goals were divided into mastery-approach goals (demonstrating competence through task mastery and personal improvement) and mastery-avoidance goals (avoiding task-referential or self-referential incompetence), while performance-approach and performance-avoidance goals were identical to those used in the trichotomous model. In the driving domain, drivers who want to master driving tasks and improve their driving skills pursue mastery-approach goals and drivers who want to avoid being a bad driver or to avoid regressing as a driver pursue mastery-avoidance goals. Once again, measures were quickly developed in the education [18] and sports [19] domains. Studies using the previous scales assessing achievement goals have provided an impressive volume of results concerning achievement motivation in the sports, education, and work domains (for reviews, see [7, 20, 21]). But studies focusing on achievement goals in driving were lacking.

However, examining achievement goals seems relevant in the driving context for several reasons. First, the driving context may be considered an achievement context. In an achievement context, personal competence is implicated and evaluated, the result depends on the individual, and success is both uncertain and socially valued [22]. The driving context meets all these conditions: the driver’s personal competence is involved in order to be effective in this domain; personal competence is continuously evaluated by the driver, by passengers, by other drivers, and/or by family and friends; the result depends on driving behaviors; success is uncertain (even a good driver can have an accident); and being a good driver is socially valued. Secondly, we focused in the present study on the 2 x 2 model of achievement goals because this model was the most used in the literature in the sports, education, and work domains [21]. Consequently, the results in the driving domain may be more easily compared with those found in these three domains. Thirdly, a driver may want to master the driving task and to improve his or her driving skills (mastery-approach goals), to outperform other drivers (performance-approach-goals), to avoid doing poorly relative to driving task demands, to avoid regressing as a driver, and to avoid being a bad driver (mastery-avoidance goals), and to avoid being a worse driver than others (performance-avoidance goals). Fourthly, the achievement goals literature has followed the same process during the past 30 years: a measure of achievement goals was first developed in a specific domain and was then adapted in another [23]. Consequently, developing and validating a scale assessing the four achievement goals in driving seemed promising to extend the achievement goal literature in another hitherto unexplored domain. This was a first step to then investigate the potential relationships between achievement goals in driving and many variables of interest in the driving domain.

Consequently, the purposes of the present study were (a) to develop a version of the Achievement Goal Questionnaire in Driving (AGQ-D); (b) to test with car drivers the factorial structure of the scale which was created; (c) to compare the 2 x 2 model (baseline model) with five alternative models; (d) to test the measurement invariance of the scale across gender; and (e) to study its concurrent validity using both key variables in the achievement goal literature (i.e., interest and self-efficacy) and self-reported variables which are relevant in the domain of driving (i.e., accidents, at-fault accidents, emergency maneuvers, and fines). Traffic accidents are particularly interesting to investigate because they are a major safety issue [24]. Car drivers were selected in the present study because they are the most represented among road users.

Based on the achievement goal literature in the education, sports, and work domains, several hypotheses can be formulated. Since analyses of factorial invariance have shown that the 2 x 2 model of achievement goals was considered as equivalent across gender in the sports domain [25] and in the education domain [26], we hypothesized that this would also be the case in the driving domain. Concerning the concurrent validity of the scale, many results have already been highlighted in the literature focusing on the sports, education, and work domains [7, 20, 21, 27]. First, interest represents a person’s enjoyment of an activity for its own sake. In the driving domain, interest represents the driving pleasure reported by drivers themselves. In the literature referred to above, interest was almost systematically positively related to mastery-approach goals, often positively related to performance-approach goals, and negatively related to avoidance-based goals [23]. Consequently, we predicted that approach-based goals and avoidance-based goals in driving were respectively positive and negative predictors of interest in driving. Secondly, self-efficacy may also be a promising variable to study. It is defined as “beliefs in one’s capabilities to mobilize the motivation, cognitive resources, and courses of action needed to meet given situational demands” (p. 408) [28]. In the driving domain, self-efficacy is the self-evaluation that drivers may have of their own driving skills in order to meet the requirements of the driving task. In the achievement goals literature, self-efficacy was mainly positively related to approach-based goals [29], whereas the pattern was not clear for avoidance-based goals. Perceived competence, strongly linked to self-efficacy, was considered a predictor of achievement goals [27]. Consequently, we hypothesized that self-efficacy in driving was a positive predictor of mastery-approach and performance-approach goals in driving. Thirdly, achievement goals were studied in relation with self-reported variables of interest in the domain of driving (i.e., accidents, at-fault accidents, emergency maneuvers, and fines). Since performance-approach goals have negative consequences in the social and ethical domains [30], we hypothesized that adopting performance-based goals may lead drivers to declare more accidents, at-fault accidents, emergency maneuvers, and fines. Since mastery-approach goals were mostly linked to adaptive outcomes in the literature [7], we hypothesized that mastery-approach goals were negative predictors of these variables. Since performance-avoidance goals were almost systematically related to maladaptive outcomes [7], we hypothesized that they were positive predictors of these variables. Finally, no hypothesis was formulated for mastery-avoidance goals due to the contrasted results found in the literature (for a review, see [31]).

## Methods

### Measure development

Following the procedure of Conroy et al. [19], the development of the scale assessing achievement goals in driving was based on Riou et al.’s French scale assessing achievement goals in sports, physical education and physical activity [32]. The French Achievement Goals Questionnaire for Sports and Exercise (FAGQSE) was revised for applicability to the driving domain. In a first step, the opening sentence was modified, replacing *“In sports*,*…”* with *“When driving*,*…”*. In a second step, the authors checked that the formulation of the 12 items of the FAGQSE was compatible with the driving domain, resulting in some wording adjustments. Riou et al.’s scale [32] was selected here for several reasons: (a) this questionnaire is the most recent 2 x 2 scale validated in French assessment of achievement goals; (b) it includes the recommendations of Elliot and Murayama about the measurement of achievement goals [33]; and (c) sports and driving may be compared based on the motor, cognitive, and perceptive skills that are involved in these two domains. Consequently, twelve items were retained, representing mastery-approach, performance-approach, mastery-avoidance, and performance-avoidance goals, with three items per subscale. The twelve items of the final questionnaire are presented below in Table 3.

### Participants and procedure

A total of 420 French car drivers (217 women, 203 men, *M*_*age*_ = 42.35 years, *SD* = 14.75, range = 18–79 years) participated in the study. Table 1 shows the number of participants for each age group. Only participants holding a category B driver’s license (i.e., the European license necessary to drive motor vehicles weighing less than 3,500 kilos) were included in the study (*M*_*years of driving license*_ = 23.03 years, *SD* = 14.66), with an annual mileage of approximately 17,000 kilometers (*SD* = 14,089.78 kilometers). Concerning driving frequency, 74.76% of the participants declared that they drove every day. 15.24% drove at least three times per week and 10% drove less than three times per week.

**Table 1:** Numbers by gender for each age group.

Gender	Age group					Total
	18–29	30–39	40–49	50–60	>60	
**Men**	49	32	56	37	29	203
**Women**	64	27	54	43	29	217
**Total**	113	59	110	80	58	420

The number of accidents since the participants had obtained their driving license ranged from 0 (28.1% of the participants) to more than 4 (11.7%), with 25% of the sample having had one accident, 23.8% two accidents, and 11.4% three accidents. The number of at-fault accidents since they had obtained their driving license ranged from 0 (50.5%) to more than 4 (3.1%), with 31% of the sample having had one at-fault accident, 11.4% two at-fault-accidents, and 4% three at-fault accidents. The number of emergency maneuvers they had made in the previous week ranged from 0 (59.5%) to more than 3 (11.4%), with 16.5% of the sample having made one emergency maneuver and 12.6% two emergency maneuvers. Finally, the number of fines and penalty points in the last year ranged from 0 (65%) to more than 3 (4%), with 22% of the sample having had one fine or penalty point and 9% two fines or penalty points.

Participants completed a questionnaire containing the focal constructs in individual Web-based or paper-based sessions. Web-based data collection and paper-based questionnaires were used to have the most diversified sample possible with respect to the participants’ ages and geographical locations. Web-based and paper-based data collection led to similar results those in in previous studies [34, 35]. In paper-based sessions, participants voluntarily and individually filled out the questionnaire without interaction with the researcher or other participants. In Web-based sessions, nonprobability snowball sampling was used as a validated research tool to recruit participants [36]. In a first step, 100 participants were contacted through social networking sites (e.g., LinkedIn). A brief description of the study (i.e., a study focusing on the psychological characteristics of car drivers), the time to complete it (about 5 minutes), and a link to complete the survey were provided. In a second step, participants were requested to forward the questionnaire link to at least three other individuals of their own social network. The only inclusion criterion was having a car driver license.

The study met the requirements of the institutional board of Aix-Marseille University and of the *Commission Nationale de l’Informatique et des Libertés* (n°2004–801). It was conducted in accordance with the Declaration of Helsinki. In Web-based and paper-based sessions, informed consent was obtained from the participants before they filled out the questionnaire. They were assured that their participation in the study would remain completely anonymous.

### Measures

#### Achievement goals in driving

The four achievement goals in driving were assessed through the scale specifically created for this study, namely the AGQ-D, using a 1 (completely disagree) to 5 (completely agree) scale. Factorial structure and internal consistency are presented in the Results section.

#### Interest in driving

This variable was assessed with an adaptation to the driving domain of Durand, Cury, Sarrazin, and Famose’s French translation of the Intrinsic Motivation Inventory [37]. Participants responded to the four items (e.g., *“I enjoy driving”*) on a 1 (strongly disagree) to 5 (strongly agree) scale. A Confirmatory Factor Analysis (CFA) was conducted using the JASP software (version 0.10). Following the recommendations of Byrne [38], Blunch [39], and Hu and Bentler [40], which are presented in detail below in the Data Analyses section, the fit statistics met the criteria for a good fitting model: χ^2^(2, *N* = 420) = 2.45, *p* = .294, CFI = 1, TLI = .999, SRMR = .012, RMSEA = .023. Using McDonald’s omega (see Data Analyses section), internal consistency was good (*ω* = .88).

#### Self-efficacy in driving

Participants’ self-assessments of their driving were measured with the French version of the driving self-efficacy scale [41], initially validated in English [42]. Because Boccara et al.’s scale was designed for learner drivers [41], two items focusing on the driving license test and driving lessons were deleted. Consequently, participants responded to the ten items (e.g., *“I am good in maneuvering the car”*) on a scale ranging from 1 (certainly) to 7 (certainly not). The results of a first CFA were not satisfactory: χ^2^(35, *N* = 420) = 210.25, *p* < .001, CFI = .866, TLI = .827, SRMR = .064, RMSEA = .109. The modification indices suggested that adding an error covariance between items 1 and 2 and between items 4 and 5 would improve model fit. This was indeed the case: χ^2^(33, *N* = 420) = 122.12, *p* < .001, CFI = .932, TLI = .907, SRMR = .054, RMSEA = .080. Internal consistency was satisfactory (*ω* = .83).

#### Self-reported information

Information usually collected in traffic psychology research was finally requested from the participants: age, gender, years of driving experience, and annual mileage. Additionally, driving self-reported information was also requested: number of accidents since they had obtained their driving license, number of at-fault accidents, number of emergency maneuvers they had made in the previous week, and number of fines and penalty points in the last year. Accident and at-fault accident scores were obtained by dividing the number of accidents and at-fault accidents by the number of years since the drivers obtained their driving license to have more representative results.

### Data analyses

Concerning preliminary analyses, the dataset was first screened for missing values. Then, Mahalanobis distance at the multivariate level (*χ*^2^(9) = 27.88, *p* < .001) was used to detect gross outliers [43]. Finally, skewness and kurtosis provided an indication of univariate normality: values ≥ |2| for skewness and ≥ |7| for kurtosis signaled variables non-normal in distribution [44]. The descriptive statistics and the correlations between variables are presented in Table 2.

**Table 2:** Descriptive statistics of the final sample (without outliers), correlations between scales, internal consistency, Skewness, Kurtosis, and discriminant validity.

Variables	*M*	*SD*	1	2	3	4	5	6	7	8	9	10
1. Mastery-approach	3.21	1.21	**(.85)**									
2. Performance-approach	1.77	1.03	.26***	**(.82)**								
3. Mastery-avoidance	4.42	0.76	.33***	.03	**(.79)**							
4. Performance-avoidance	2.46	1.25	.32***	.61***	.19***	**(.75)**						
5. Self-efficacy	5.58	0.82	.00	.05	.14**	-.03	-					
6. Interest	3.84	0.98	.21***	.20***	.02	.14**	.41***	-				
7. Gender	-	-	-.01	-.04	.05	.04	.03	-.02	-			
8. Age	42.35	14.75	-.08	-.22***	.02	-.17***	-.09	-.19***	.03	-		
9. Years of driving license	23.03	14.66	-.10*	-.24***	-.01	-.19***	-.07	-.17***	.03	.95***	-	
10. Annual mileage (in km.)	16998	14089	.05	.12*	.01	.07	.08	.09	.00	-.02	-.01	-
McDonald’s omega	-	-	.85	.88	.75	.83	.83	.88	-	-	-	-
Skewness	-	-	-0.510	1.021	-0.845	0.210	-0.800	-0.820	-	-	-	-
Kurtosis	-	-	-0.021	0.404	0.750	-0.997	1.067	0.156	-	-	-	-

*p < .05

**p < .01

***p < .001, *M* = Mean, *SD =* Standard Deviation, Gender (boys = 1, girls = 0), the diagonal elements in bold represent $\sqrt{AVE}$ for the four achievement goals, AVE = Average Variance Extracted.

Concerning primary analyses focusing on the internal validity of the AQG-D, different steps were followed. In a first step, a CFA was conducted using the JASP software (version 0.10) on the covariance matrix of the items, and the solution was generated using maximum likelihood estimation. The Comparative Fit Index (CFI), the Tucker-Lewis Index (TLI), the Standardized Root Mean Square Residual (SRMR), and the Root Mean Square Error of Approximation (RMSEA) were used as fit indices in the present study. CFI ≥ .95, TLI ≥ .95, and RMSEA ≤ .05 were the criteria for a good fitting model, and CFI ≥ .90, TLI ≥ .90, and RMSEA ≤ .08 were the criteria for an acceptable fitting model [38]. A value less than .08 is generally considered a good fit for SRMR [40]. In a second step, convergent validity was tested with three procedures [45]: item reliability (each factor loading needs to be higher than .50), composite reliability (the values need to be greater than .70 for each factor), and the average variance extracted (AVE, the values need to be higher than .50 for each factor). In a third step, discriminant validity was assessed. The diagonal elements of the latent correlation matrix were replaced by $\sqrt{AVE}$. If this value is higher than the correlation between the factor and other factors of the model of interest, the items are considered independent of one another [46]. In a fourth step, McDonald’s omega was used to estimate internal consistency rather than Cronbach’s alpha, which has a higher likelihood of over- or under-estimating reliability [47]. McDonald’s omega needs to be above .70 to be considered satisfactory. In a fifth step, additional analyses were conducted to compare the fit of the 2 x 2 baseline model with five alternative models. The Akaike Information Criterion (AIC) was computed for each model and the model with the smallest AIC showed the greatest potential [38]. In a sixth and last step, we followed the procedure of Putnick and Bornstein [48] to test the gender invariance of the AGQ-D at the configural, metric, and scalar levels. Two criteria were used to validate a level: a change in CFI up to -.01 and a change in RMSEA up to .015 [49].

Concerning primary analyses focusing on the concurrent validity of the AGQ-D, two hierarchical regression analyses were conducted. The first one examined in Step 2 how the four achievement goals in driving predicted interest in driving, accidents, at-fault accidents, emergency maneuvers, and fines, controlling in Step 1 gender, age, years of driving license, and annual mileage. The second hierarchical analysis examined in Step 2 how self-efficacy in driving predicted the four achievement goals, controlling in Step 1 the same variables as in the first hierarchical regression analyses.

## Results

### Preliminary results

Only 0.01% of the data were missing, so they were replaced by the mean of the participant’s subscale [50]. Because they showed a Mahalanobis distance higher than the cut-off value of *χ*^2^(9) = 27.88, *p* < .001, two participants were detected as gross outliers and were excluded from the study. Measures of sample skewness (maximum = 1.021) and kurtosis (maximum = 1.067) showed that the distribution was approximately normal for the different variables of interest.

### Factorial structure, convergent validity, discriminant validity, and internal consistency of the AGQ-D

The results of the CFA conducted on the covariance matrix of the 12-items AGQ-D met the criteria for an acceptable fitting model: χ^2^(48, *N* = 420) = 109.56, *p* < .001, CFI = .974, TLI = .965, SRMR = .041, RMSEA = .055. Convergent validity was also supported, because standardized factor loadings ranged from .65 to .92 (item reliability), composite reliability ranged from .79 to .89, and AVE ranged from .56 to .72 (see Table 3). Moreover, discriminant validity was considered satisfactory (see Table 2), evidencing that each achievement goal shares more variance with its items than it does with other achievement goals. Finally, a good level of internal consistency was found for mastery-approach (*ω* = .85), performance-approach (*ω* = .88), mastery-avoidance (*ω* = .75), and performance-avoidance (*ω* = .83) goals.

**Table 3:** French version of the AGQ-D (and English translation), standardized factor loading, construct reliability, and average variance extracted.

Factors/Items: En conduite automobile,… *(When driving*,*…)*	Standardized factor loading	Composite reliability	Average variance extracted
***Factor 1*: *Mastery-approach goals***		.89	.72
1. Mon but est de progresser autant que possible	.75		
*(My goal is to progress as much as possible)*			
5. Mon but est de m’améliorer le plus possible	.92		
*(My goal is to improve as much as possible)*			
9. Mon but est de conduire de mieux en mieux	.77		
*(My goal is to drive better and better)*			
***Factor 2*: *Performance-approach goals***		.86	.68
2. Mon but est de surpasser les autres	.88		
*(My goal is to outperform others)*			
6. Je cherche à être au-dessus des autres	.84		
*(I am striving to be superior to others)*			
10. Mon but est d’être plus performant(e) que les autres	.83		
*(My goal is to perform better than others)*			
***Factor 3*: *Mastery-avoidance goals***		.83	.63
3. Je cherche à éviter de mal faire les choses	.77		
*(I am striving to avoid doing things badly)*			
7. Mon but est d’éviter de faire des erreurs	.69		
*(My goal is to avoid making mistakes)*			
11. Je cherche à éviter de mal conduire	.65		
*(I am striving to avoid driving badly)*			
***Factor 4*: *Performance-avoidance goals***		.79	.56
4. Je cherche à éviter d’être en dessous des autres	.82		
*(I am striving to avoid being inferior to others)*			
8. Mon objectif est d’éviter de faire moins bien que les autres	.82		
*(My aim is to avoid performing worse than others)*			
12. Mon but est d’éviter de moins bien conduire que les autres	.73		
*(My goal is to avoid driving less well than others)*			

### Comparison with alternative models

Similarly to the procedure used in several studies [18, 19], the fit of the baseline model (2 x 2 AGQ-D) was compared with five alternative models: (1) a trichotomous model whereby the mastery-based goals load on a combined latent factor and the performance-approach and performance-avoidance goals load on their hypothesized latent factors; (2) a dichotomous model (or definition model) whereby the mastery-based and performance-based goals load on two different combined latent factors; (3) an approach model whereby the approach-based items load on a combined latent factor and the avoidance-based items load on their hypothesized latent factor; (4) an avoidance model whereby the avoidance-based items load on a combined latent factor and the performance-based items load on their hypothesized latent factor; and (5) a valence model whereby the items with the same valence load together on combined latent factors. The results presented in Table 4 show that the baseline 2 x 2 model provides a better fit to the data than the trichotomous, dichotomous, approach, avoidance, and valence models.

**Table 4:** Results of the confirmatory factor analyses for the 2 x 2 model (baseline model) and for five alternative models.

	*χ^2^*	*df*	*p*	RMSEA	CFI	TLI	SRMR	AIC
**2 x 2 model**	109.56	48	< .001	.055	.974	.965	.041	13889.62
**Trichotomous model**	341.91	51	< .001	.117	.878	.843	.088	14115.97
**Dichotomous model**	537.38	53	< .001	.148	.798	.748	.103	14307.44
**Approach model**	903.02	51	< .001	.199	.644	.539	.172	14677.07
**Avoidance model**	424.90	51	< .001	.132	.844	.798	.120	14198.95
**Valence model**	958.73.61	53	< .001	.202	.621	.529	.177	14729.78

RMSEA = Root Mean Square Error of Approximation, CI = Confidence Interval, CFI = Comparative Fit Index, TLI = Tucker-Lewis Index, SRMR = Standardized Root Mean Square Residual, AIC = Akaike Information Criterion.

### Gender invariance

The AGQ-D was found to be invariant across gender at the configural level (ΔCFI = -.003, ΔRMSEA = .004), the metric level (ΔCFI = .000, ΔRMSEA = .004), and the scalar level (ΔCFI = -.001, ΔRMSEA = .001).

### Concurrent validity

Controlling gender, age, years of driving license, and annual mileage, the results of the hierarchical regression analyses highlighted that: (a) mastery-approach and performance-approach goals were positive predictors of interest in driving; (b) performance-approach goals were positive predictors of emergency maneuvers; and (c) mastery-avoidance goals were negative predictors of accidents and at-fault accidents. No significant predictions were found for fines and penalty points, or for performance-avoidance goals. The detailed results are presented in Table 5. Furthermore, a second hierarchical regression analysis showed that self-efficacy was a positive predictor of mastery-avoidance goals only (*β* = .15, *p* = .002).

**Table 5:** Summary of hierarchical regression analyses predicting interest, accidents, at-fault accidents, emergency maneuvers, and fines.

	Interest		Accidents		At-fault accidents		Emergency maneuvers		Fines	
	*R*^2^	*β*	*R*^2^	*β*	*R*^2^	*β*	*R*^2^	*β*	*R*^2^	*β*
*Step 1*:	.043**		.136***		.93***		.053***		.021	
Gender^1^		-.01		-.10*		-.09		.12*		.05
Age^1^		-.20		.04		.14		-.02		-.17
Years of driving license^1^		.06		-.37**		-.42**		-.11		.08
Annual mileage^1^		.07		.01		-.02		.09		.11*
*Step 2*	.094***		.150***		.117***		.083***		.028	
Mastery-approach goals		.19***		.09		.02		.03		-.03
Performance-approach goals		.12*		.05		-.05		.18**		.03
Mastery-avoidance goals		-.04		-.10*		-.13**		-.07		-.01
Performance-avoidance goals		-.01		-.02		.04		-.04		-.08

*p < .05

**p < .01

***p < .001

^1^The *β* coefficients from the final regression equation

## Discussion

This study is the first to apply the 2 x 2 model of achievement goals to the driving domain. It showed the good psychometric properties of the 12-items measure (Achievement Goal Questionnaire in Driving, AGQ-D); the better fit of this model compared to five other models; the gender invariance of the AGQ-D; and its concurrent validity using both variables central in the achievement goal literature (interest and self-efficacy) and in the driving literature (accidents, at-fault accidents, emergency maneuvers, and fines). The AGQ-D is now a tool available to examine more precisely the relationships between achievement goals in driving and other variables of interest in the driving domain (e.g., risk-taking, objective accidents, driving errors, positive driving behaviors) and with other procedures such as longitudinal designs.

As expected, performance-approach and mastery-approach goals were positive predictors of interest in driving, which was consistent with most of the studies in sports [51] and education [52]. Interest is one of the most important key variables in research focusing on achievement motivation, representing the interest and enjoyment experienced by an individual involved in an activity for its own sake [53]. Because approach motivation is an appetitive form of motivation, these results of the present study in the driving domain were not surprising, especially for mastery-approach goals [23]. As expected, performance-approach goals were also positive predictors of self-reported emergency maneuvers. Performance-approach goals have negative consequences in the social and ethical domains [20, 21, 30], especially a lack of interest in the rules [54]. Outperforming others is the key factor for drivers who adopt performance-approach goals and want to show their superiority to other drivers. Consequently, they may take greater risks than drivers adopting other achievement goals and this risk-taking may result in more frequent emergency maneuvers. For example, a driver who does not stop at a red light so as to overtake other cars or a driver who exceeds the speed limit to drive faster than other drivers is more likely to make emergency maneuvers than drivers who respect these rules. But these relationships between achievement goals and risk-taking are hypothetical. Consequently, these assumptions need to be tested using specifically the aggressive violations and ordinary violations subscales of the Driving Behavior Questionnaire [55], recently validated in French [56].

More surprisingly, mastery-avoidance goals negatively predicted self-reported accidents and at-fault accidents, which identify for the first time a potential protective role of these goals in the driving domain yet to be confirmed. This result is consistent with the fact that mastery-avoidance goals were also positively predicted in the present study by driving self-efficacy, evidencing that the drivers with high perceived ability were more likely to adopt mastery-avoidance goals. Mastery-avoidance goals are avoidance-based goals, and theoretically avoidance motivation produces more maladaptive effects than approach motivation [12]. But mastery-avoidance goals are a combination of positive (mastery) and negative (avoidance) elements [57]. Theoretically, adaptive or maladaptive outcomes could be produced depending on which of the two components is the more predominant [8]. While some studies have called into question the interest of the mastery-avoidance goal construct [58], Van Yperen, Elliot, and Anseel [59] showed that mastery-avoidance goals were the most important goals for 15% of individuals in the sport domain, 33% in the education domain, and 49% in the work domain, evidencing that mastery-avoidance goals were key elements to consider in achievement contexts. The meta-analysis of Baranik et al. [31] and the study of Senko and Freund [57] showed that mastery-avoidance goals were positively related to both adaptive variables (interest, need for achievement, perceived competence) and maladaptive variables (negative affect, competitiveness, anxiety, procrastination, maladaptive forms of perfectionism, ineffective task strategies). They were also negatively related to performance and help-seeking, evidencing that they are quite detrimental, even if they are less dysfunctional than performance-avoidance goals. Identifying promising perspectives, Baranik et al. [31] called for the examination of other antecedents and consequences of mastery-avoidance goals toward a better understanding of this construct. In the present study, they were negative predictors of self-reported accidents and at-fault accidents. We postulate that these results could be explained by drivers’ fear of failure, which may be specific to the driving domain. Fear of failure is the motive to avoid failure which was considered by the early work of Murray [60] to be an energizing agent affecting human behavior, especially in an achievement context. In the education [18] and sports [19] domains, mastery-avoidance goals have been positively related to fear of failure. In the driving domain, failure has a special status because it may induce accident, injury, and even death. For example, overtaking a car in a bend without any visibility strongly increases the probability of being struck by another car. Consequently, the fear of failure in the driving domain–especially the fear of the consequences of failure–may lead some drivers to adopt mastery-avoidance goals (i.e., striving not to do poorly relative to driving task demands, avoiding being a bad driver) and to avoid risky driving behaviors that may result in accidents with consequences that would be more or less dramatic. However, this explanation is speculative and needs to be tested in future studies including fear of failure measures.

Surprisingly, mastery-approach goals were found to be positive predictors only of interest in driving, whereas in the achievement goal literature they are the strongest predictors of adaptive outcomes (for reviews, see [7, 20, 21]). Van Yperen et al. [59] showed that three patterns of results were found when studying the relationships between mastery-approach goals and performance in contexts in which performance-based goals are relevant: positive relationships, null effects, and detrimental effects (due to overemphasis on mastery). No oversized significant effects were found in the driving domain, contrary to expectations. Moreover, the process model of Senko and Harackiewicz [61] identified that the perception of the difficulty of achievement goals may influence outcomes. A goal which seems to be hard to attain may induce performance pressure and may influence perceived competence or performance. In the present study, the mean of participants’ mastery-approach goals was lower than the mean of their mastery-avoidance goals. Consequently, it may be harder for drivers to adopt mastery-approach goals (focusing on improving one’s own driving competence and on mastering the driving task) than mastery-avoidance goals (focusing on avoiding regressing and on striving not to do poorly relative to the demands of the driving task, for example, avoiding driving mistakes). Identifying in future studies the perceived goal difficulty [57] may assist in testing the previous assumption in the driving domain.

Several limitations may be observed in the present study. First, a test-retest procedure was not conducted on the AGQ-D and the studies took place in a single country (France). Because driving behaviors may differ across countries [62], cross-cultural studies would be necessary to identify whether mastery-avoidance goals may also be prevalent in other countries in negatively predicting self-reported accidents and at-fault accidents. Secondly, sample size could be increased in large-scale studies to have the opportunity to examine the results according to several age groups. For example, Martinussen, Lajunen, Møller, and Özkan’s study [63] included 4335 participants and seven age groups (18–24 years, 25–34, 35–44, 45–54, 55–64, 65–74, 75–84). Thirdly, drivers’ behaviors were self-reported. Future studies using the AGQ-D might focus on objective indicators such as those used in Naturalistic Driving Studies using measures of drivers’ behaviors in the real world through data acquisition systems located in the vehicles [64].

Complementarily to the perspectives previously identified with the 2 x 2 model of achievement goals, adapting the 3 x 2 model of achievement goals to the driving domain may also be relevant. Elliot, Murayama, and Pekrun [65] recently validated the 3 x 2 model, bifurcating mastery-based goals into task-based (satisfying or not the absolute demands of the task) and self-based (improving or regressing relative to own’s trajectory) goals. This scale, already used in the education [65, 66] and sports [23] domains, may be a promising perspective toward a deeper understanding of achievement goals in driving. Because mastery-avoidance goals were the strongest predictors of accidents and at-fault accidents, separating mastery-avoidance goals of the 2 x 2 model in task-avoidance goals (avoiding driving task failure) and self-avoidance goals (not driving worse than before) in the 3 x 2 model may provide information about the definition of mastery-based goals which may be the most prevalent in the driving domain (task and/or self). Finally, the present study has only included car drivers. Because representations of risk factors are different between car drivers and two-wheeler drivers [67], conducting a study focusing on achievement goals of two-wheeler riders may be relevant to identify if the pattern of achievement goals is the same as for car drivers and to examine the predictive role of achievement goals on self-reported and objective risky behaviors of two-wheeler riders.

## Conclusions

Based on previous studies focusing on other achievement contexts (e.g., education, sports, work) which have provided an extensive literature, a questionnaire, namely the Achievement Goals in Driving (AGQ-D) questionnaire, was developed in the present study to assess achievement goals in the driving domain. This scale was validated using a confirmatory analysis and was compared with five alternative models. The questionnaire also showed its gender invariance. The AGQ-D is now a scale available to start and develop research on achievement goals in the driving domain. Investigating achievement goals in the driving domain is worthwhile to consider in order to both better understand drivers’ psychological characteristics and to increase road safety. In the present study, achievement goals in driving were related to self-reported accidents, at-fault accidents, and emergency maneuvers. In other studies, performance-based goals were positively related with rule violations and aggressive behaviors in other domains [68] and strong association was found between risky behaviors and traffic accidents [4]. Consequently, the potential role of achievement goals in driving in explaining risky behaviors is a promising avenue for research in the driving domain.

## Supporting information

S1 File Anonymized data set used in the study. (XLSX)
